# High-mobility group box 1 protein (HMGB1) from Cherry Valley duck mediates signaling pathways and antiviral activity

**DOI:** 10.1186/s13567-020-00742-8

**Published:** 2020-02-18

**Authors:** Xiaolan Hou, Gen Liu, Huihui Zhang, Xiaofang Hu, Xinyue Zhang, Fei Han, Huizhen Cui, Jinjian Luo, Ru Guo, Rong Li, Ning Li, Liangmeng Wei

**Affiliations:** 1 College of Animal Science and Veterinary Medicine, Sino-German Cooperative Research Centre for Zoonosis of Animal Origin of Shandong Province, Shandong Provincial Key Laboratory of Animal Biotechnology and Disease Control and Prevention, Shandong Provincial Engineering Technology Research Center of Animal Disease Control and Prevention, Shandong Agricultural University, 61 Daizong Street, Tai’an, 271018 Shandong China; 2 Collaborative Innovation Center for the Origin and Control of Emerging Infectious Diseases, Shandong First Medical University, Tai’an, 271000 Shandong China; 3 Key Laboratory of Precision Oncology of Shandong Higher Education, Institute of Precision Medicine, Jining Medical University, Jining, 272067 Shandong China

## Abstract

High-mobility group box 1 protein (HMGB1) shows endogenous damage-associated molecular patterns (DAMPs) and is also an early warning protein that activates the body’s innate immune system. Here, the full-length coding sequence of HMGB1 was cloned from the spleen of Cherry Valley duck and analyzed. We find that duck HMGB1(duHMGB1) is mostly located in the nucleus of duck embryo fibroblast (DEF) cells under normal conditions but released into the cytoplasm after lipopolysaccharide (LPS) stimulation. Knocking-down or overexpressing duHMGB1 had no effect on the baseline apoptosis rate of DEF cells. However, overexpression increased weakly apoptosis after LPS activation. In addition, overexpression strongly activated the IFN-I/IRF7 signaling pathway in DEF cells and significantly increased the transcriptional level of numerous pattern recognition receptors (PRRs), pro-inflammatory cytokines (IL-6, TNF-α), IFNs and antiviral molecules (OAS, PKR, Mx) starting from 48 h post-transfection. Overexpression of duHMGB1 strongly impacted duck virus replication, either by inhibiting it from the first stage of infection for novel duck reovirus (NDRV) and at late stage for duck Tembusu virus (DTMUV) or duck plague virus (DPV), or promoting replication at early stage for DTMUV and DPV infection. Importantly, data from duHMGB1 overexpression and knockdown experiments, time-dependent DEF cells transcriptional immune responses suggest that duHMGB1 and RIG-I receptor might cooperate to promote the expression of antiviral proteins after NDRV infection, as a potential mechanism of duHMGB1-mediated antiviral activity.

## Introduction

High-mobility group box 1 protein (HMGB1) belongs to a family of nonhistone chromosomal proteins, which are widely conserved in the nucleus of eukaryotic cells. HMGB1 was discovered in the 1960s and was named for its high migration ability in polyacrylamide gel electrophoresis [1]. HMGB1 has two N-terminal DNA-binding domains—HMG box A and box B—as well as an acidic C-terminal domain [2].

In mammals, HMGB1 has two nuclear localization sequences and no endoplasmic reticulum localization sequence; thus, HMGB1 is normally located in the nucleus. Proper signal stimulation leads to high acetylation of HMGB1 resulting in cytosolic relocation [3]. HMGB1 has different redox states due to the different extracellular redox environment. HMGB1 in the all-thiol state acts primarily on the RAGE receptor resulting in the production and release of pro-inflammatory cytokines and chemokines [4]. When presented in the oxidative environment, cysteines 23 and 46 in the HMGB1 A box form a sulfide bond effectively producing the disulfide form of HMGB1. This disulfide HMGB1 can act on the TLR4 receptor and modulate the production of inflammatory cytokines [5, 6].

Studies have confirmed that the HMGB1 from humans can be involved in inflammatory responses as a proinflammatory cytokine [7]. HMGB1 is an endogenous damage-associated molecular patterns (DAMPs) biomolecule. At the onset of inflammation, HMGB1 can be passively released from necrotic cells or actively secreted by stimulated monocytes/macrophages. The release of HMGB1 is observed later than the pro-inflammatory mediators, such as IL-1β and TNF-α, but is rather sustained. Thus, HMGB1 is considered to belong to the late inflammatory mediators in rats [8, 9].

HMGB1 has cytokine-related characteristics and can be actively secreted by activated immune cells (such as monocytes/macrophages, natural killer cells, and dendritic cells). It acts on the surface receptors of immune cells and endothelial cells. Extracellular HMGB1 induces the expression of inflammatory factors and further release of HMGB1 which leads to exacerbation of inflammation. HMGB1 stimulates the release of chemokines and cytokines, and increases the expression of adhesion molecules involved in immune responses, thus inducing the chemotaxis and activation of inflammatory cells and favouring the disruption of the epithelial barrier [10, 11].

Recent studies have shown that the DAMPs are released after cell damage or death, which become new hotspots in the initiation and persistence of innate immune responses [12]. HMGB1 is involved in the pathogenesis of a variety of viral diseases. Cellular HMGB1 and “replication transcriptional activator” (Rta) synergistically up-regulate the ORF 50 promoter to promote Kaposi’s sarcoma-associated virus replication [13]. Extracellular HMGB1 is a late inflammatory mediator released after infection with West Nile virus [14], atypical pneumonia virus [15], porcine reproductive and respiratory syndrome virus [16], grass carp reovirus [17]. However, while the involvement of HMGB1 in a variety of viral diseases has been confirmed, the presence or absence of HMGB1 in ducks and the best approaches to regulate the host’s antiviral innate immune mechanisms are currently unclear.

## Materials and methods

### Animals, cells, virus, and ligands

Cherry Valley ducks were purchased from a farm near Taian, China. Duck embryo fibroblast (DEF) cells derived from 11-day-old duck embryos were cultured in Dulbecco’s modified Eagle medium (DMEM) (Gibco, Grand Island, NY, USA) with 10% fetal bovine serum (Transgen, Beijing, China). These samples were cultured at 37 °C, 5% (v/v) CO_2_. Duck Tembusu virus (DTMUV)-FX2010 strain, novel duck reovirus (NDRV), and duck plague virus (DPV)-GM strain were used in this study, as described [18–21]. DEF cells were first seeded into 96-well plates and used when the cells reached 80% confluency. Ten-fold dilutions of the virus stock solution were prepared in DMEM medium and 100 μL of each dilution was added to a 96-well cell culture plate; eight replicates were set for each dilution. A blank cell culture control was also set up. The cells were cultured in a 37 °C, 5% (v/v) CO_2_ incubator. Finally, the cells were observed each day until the CPE produced by the virus no longer progressed. The virus titers were determined to be 10^4.9^ (DTMUV), 10^4.2^ (NDRV), and 10^6.1^ (DPV) TCID_50_ (50% tissue culture infective dose)/mL in DEF cells by the Reed and Muench method [22]. Lipopolysaccharide (LPS) from *Escherichia coli* O111:B4 and purified by phenol extraction was purchased from Sigma (Sigma-Aldrich Corp., St. Louis, MO, USA).

### Molecular cloning of the HMGB1

Total RNA was extracted from duck spleen via TransZol up (Transgen). Reverse transcription of RNA into cDNA used a HiScriptRII One Step RT-PCR kit (Vazyme, Nanjing, China). To clone the duck HMGB1 (duHMGB1), primers (Additional file 1) were designed based on the predicated gene in the GenBank (Accession Number, XM_027469875.1) (Additional file 2).

All PCR products were analyzed using electrophoresis on a 1% agarose (Biowest, Hong Kong) gel in 1 × TAE at 120 V for 20 min. The PCR products were then cloned into a pMD19-T (TaKaRa) vector and transformed into *E. coli* DH5α (Vazyme, Nanjing, China). Competent cells were then sequenced.

### Animal experiments

Three-week old healthy ducks were used as source of lymphatic, circulatory, digestive, respiratory, urinary, and central nervous tissues including the bursa, spleen, heart, glandular stomach, intestine, trachea, lung, kidney, brain, etc. The extraction and reverse transcription of total RNA were performed as described above. The expression of duHMGB1 in these tissues and organs was measured using a SYBR Green PCR Kit (Vazyme, Nanjing, China).

### Plasmid construction

The DNA fragment containing the complete ORF of duHMGB1 to which the *BamH* I and *Not* I restriction sites were added was subcloned into the pcDNA3.0(+) expression vector using Hieff Clone^TM^ Multi One Step Cloning Kit (Yeasen, Shanghai, China). This recombinant plasmid was named pcDNA3.0(+)-duHMGB1-Flag.

### Western blotting analysis

DEF cells were cultured in a 6-well plate for 12–24 h. When the cells reached approximately 80% confluence, the pcDNA3.0(+)-duHMGB1-Flag and pcDNA3.0(+)-Flag were transfected into the DEF cells using Lipofectamine 2000 (Invitrogen, Carlsbad, CA, USA), respectively. After 24 h, the cells were lysed with RIPA buffer (Solarbio, Beijing, China) containing protease inhibitor (Beyotime). The processed protein samples were subjected to SDS-PAGE electrophoresis, and the proteins were transferred to polyvinylidene fluoride (PVDF) membrane (Solarbio, Beijing, China). The PVDF membrane was blocked with 5% skim milk powder overnight at 4 °C. The samples were then incubated with mouse anti-Flag antibody (ProteinTech, Shenzhen, China) for 2 h at 37 °C. The membrane was then incubated with the secondary antibody under similar conditions. The protein bands were visualized with an ECL kit (Bio-Rad).

### Indirect immunofluorescence

DEF cells were seeded in 24-well culture plates plated with cell-climbing slices. The pcDNA3.0(+)-duHMGB1-Flag was transfected into DEF cells as an experimental group, and pcDNA3.0(+)-Flag was transfected into DEF cells as a control group. Subcellular localization of duHMGB1 was determined at 24 hours post-transfection (hpt).

We next studied duHMGB1 release into the cytoplasm upon LPS-stimulation. After transfecting pcDNA3.0(+)-duHMGB1-Flag into DEF cells for 24 h, 500 ng/mL LPS was added to the experimental group, and the control group was treated with equal volumes of DMEM medium. Immunofluorescence imaging of DEF cells was performed at 12, 24 and 36 h after LPS treatment.

Cells were fixed with 4% paraformaldehyde for 15 min and then permeabilized to the cell membrane for 10 min with 0.1% Triton X-100. The cells were incubated with mouse anti-Flag antibody (ProteinTech, Shenzhen, China) for 1 h at 37 °C, and then incubated with fluorescein isothiocyanate (FITC)-goat anti-mouse IgG (Transgen) at 37 °C for 45 min. Finally, the cell climbing slices were taken out. The cells were studied with a laser scanning confocal microscope after sealing with mounting medium (DAPI antifade, Solarbio).

### RNA interference

Three interfering RNA-targeting HMGB1 sequences were purchased from GenePharma (Shanghai, China); the sequence of the synthesized small interfering RNA is as follows: Si-duHMGB1-1, sense 5′-GGCUGACAAGCUUCGUUAUTT -3′, antisense 5′-AUAACGAAGCUUGUCAGCCTT-3′; Si-duHMGB1-2, sense 5′-GCAGAUGAUAAACAGCCUUTT-3′, antisense 5′-UUCAAACUUCCCUUUCUCCTT-3′. Si-duHMGB1-3, sense 5′-GCAGAAAGGGAAGUUUGAATT-3′, antisense 5′-UUCAAACUUCCCUUUCUCCTT-3′; and Si-NC, sense: 5′-UUCUCCGAACGUGUC ACGUTT-3′ antisense: 5′-ACGUGACACGUUCGGAGAATT-3′. Three interfering RNA and control (NC) interfering RNA were transfected into DEF cells using Lipofectamine 2000 (Invitrogen, Carlsbad, CA, USA). The DEF cells were seeded in 6-well plates and transfected with 2 μg/well of siRNA. Their interference efficiencies were analyzed by quantitative real-time PCR (qRT-PCR) after 36 h of transfection.

### Flow cytometry

DEF cells were seeded at a density of 1 × 10^6^ cells per well into 12-well plates and cultured overnight at 37 °C. To investigate whether duHMGB1 affects apoptosis in DEF cells, two sets of experiments were designed. In the first set of experiments, DEF cells were transfected with pcDNA3.0(+)-duHMGB1-Flag or pcDNA3.0(+)-Flag (control group). The apoptosis rate of DEF cells was examined for 48 h after transfection. In the second experiment, pcDNA3.0(+)-duHMGB1-Flag and pcDNA3.0(+)-Flag were transfected into DEF cells. After 24 h, 500 ng/mL LPS was added and the culture was continued for 24 h to determine the apoptosis rate. All cells were digested with trypsin (without EDTA), and the digestion was stopped with complete medium. The apoptosis rate of the DEF cells was measured with a flow cytometer using a FITC Annexin V Apoptosis Detection Kit (BD Biosciences, Franklin Lakes, NJ, USA).

### Detection of related gene mRNA expression levels

The DEF cells were cultured in a 6-well plate for 12–24 h. The pcDNA3.0(+)-duHMGB1-Flag and pcDNA3.0(+)-Flag were transfected into the DEF cells using Lipofectamine 2000 (Invitrogen, Carlsbad, CA, USA) when the cells reached approximately 80% confluence. Cells from the experimental group (pcDNA3.0(+)-duHMGB1-Flag) and the control group (pcDNA3.0(+)-Flag) were collected at each time point at 24, 36, 48, and 60 hpt. The qRT-PCR was performed using ChamQ^TM^ SYBR^®^ qPCR Master Mix (Vazyme, Nanjing, China) to detect the relative expression of target genes with primer sequences in Additional file 3. The duck glyceraldehyde-3-phosphate-dehydrogenase (GAPDH, GenBank ID: GU564233.1) was used as an endogenous reference gene. The fold-changes in gene expression were calculated using the 2^−ΔΔCT^ method with GAPDH serving as a normalization gene and mean control values as the baseline reference. [23] The differences among the groups were evaluated by non-parametric tests (Mann–Whitney U tests) using SPSS software version 17.0 (SPSS Inc., Chicago, IL, USA), *P < 0.05; **P < 0.01; ***P < 0.001.

### Dual-luciferase reporter assay

DEF cells in 24-well plate with 80% confluence were co-transfected with pcDNA3.0(+)-duHMGB1-Flag plasmid or empty vector (500 ng/well), reporter plasmid (100 ng/well), and pRL-TK plasmid (Promega) (50 ng/well) by Lipofectamine 2000 (Invitrogen, Carlsbad, CA, USA). The luciferase reporter plasmids (pGL3-IFN-β-Luc, pGL3-IRF7-Luc and pGL3-NF-κB plasmids) were prepared in-house [18, 24]. The specific details are as follows: The promoter of the avian (chicken) for IFN-β was CCTCCAGTACAGCCACCACATGGTCTCACCTTGCCAGACTCAAGAGAAGCCTGAAGGAAAAAAGCAAATAGAAAGCAAAACGAAAAATGGAAACAAGGGAATTCTCTCTACATAATGATGAAAAGAAACATGCAACATCTCATAAAGCTGGCCTCACTGCAACACCCCAAAC. The chicken IRF-7 (chIRF-7) binding positive regulatory domains were predicted by the TFSEARCH: Searching Transcription Factor Binding Sites. The pGL3-chIRF-7-Luc contains four copies of the IRF-7-positive regulatory domain motif of the chicken IFN-β promoter in front of a luciferase reporter gene (sequence: TTCACTTTCAATA). Cells were harvested as lysate at four time points, and the luciferase activity was detected with a dual-luciferase reporter assay system (Promega) according to the manufacturer’s instructions.

### Detection of antiviral activity of duHMGB1

The pcDNA3.0(+)-duHMGB1-Flag and empty vector were transfected into DEF cells at 80% confluence in 6-well plates. Cells transfected with pcDNA3.0(+)-duHMGB1-Flag served as the experimental group, and cells transfected with an empty vector were the control group. All cells were infected with DTMUV, NDRV, and DPV at 24 hpt, respectively. The medium in the 6-well plate was discarded, and the cells were washed with PBS three times, followed by infection with the viruses at the 10 TCID_50_/mL concentration for 1 h. The virus solution was then discarded, and cells were washed with PBS twice, and 2 mL low-serum medium (DMEM with 2% fetal bovine serum) was added into each well.

The Si-duHMGB1-2 and Si-NC were transfected into DEF cells at 70% confluence in 6-well plates. Cells transfected with Si-duHMGB1-2 served as the experimental group, and cells transfected with an Si-NC served as control group. The medium in the 6-well plate was discarded after 36 h, and the cells were washed with PBS for three times, followed by infection with 1 TCID_50_/mL NDRV for 1 h. The virus solution was then discarded, and the cells were washed with PBS twice, and 2 mL low-serum medium (DMEM with 2% fetal bovine serum) was added into each well.

At 12, 24, 36, and 48 hours post-infection (hpi), culture supernatants were collected for RNA and viral DNA extraction. RNA extraction and reverse transcription were conducted as described above. Viral DNA was extracted using viral DNA kits (Omega, CA, USA) according to the manufacturer’s instructions.

### Statistical analysis

All data were expressed as mean ± SE of three independent experiments. Significance was determined with the Mann–Whitney U tests using SPSS software version 17.0 (SPSS Inc., Chicago, IL, USA). *P* values less than 0.05 were considered indicative of statistical significance.

## Results

### Cloning, structure, and phylogenetic analysis of duHMGB1

**Figure 1:**
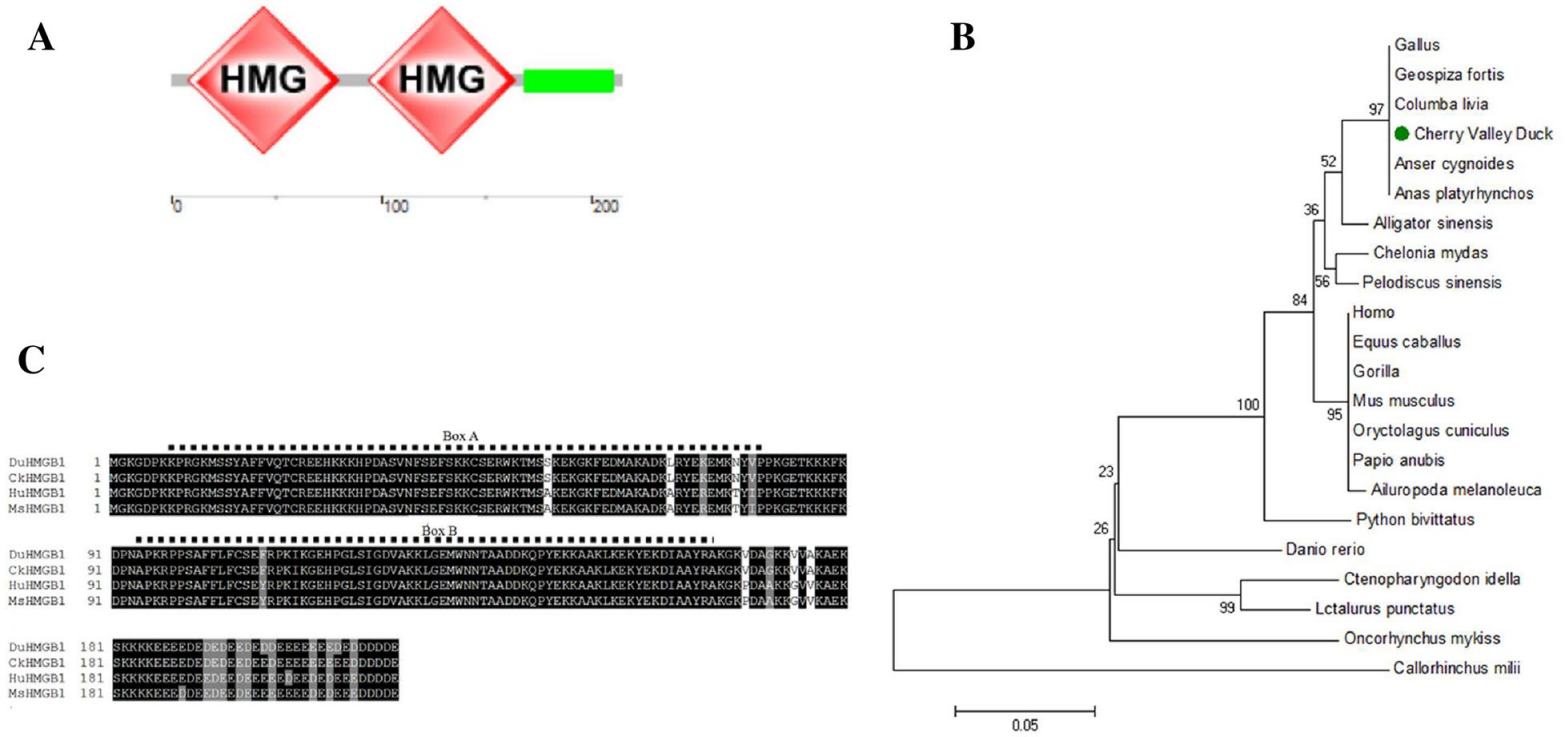
**Characterization of duHMGB1**. **A** Protein motifs of duHMGB1 were analyzed using SMART. **B** Amino-acid alignment of duHMGB1. Alignment was performed using the Clustal X program and edited with Boxshade. HMGB1 sequences are shown for the Cherry Valley duck (Du), chicken (Ch), human (Hu), and mouse (Mu). Black shading indicates amino acid identity, gray shading indicates similarity (50% threshold). **C** A phylogenic tree based on duHMGB1 the amino acid sequences of the Cherry Valley duck and other species. The neighbor-joining tree was generated using MEGA 7.0, and a 1000-replicate bootstrap analysis was performed. Scale bar is 0.05. GenBank accession numbers are shown in Additional file 2.

Two pairs of primers were designed with reference to NCBI’s duck HMGB1 predicted sequence (accession number, XM_027469869), and 892 bp and 710 bp sequences were obtained, respectively, to obtain HMGB1 intact CDs sequence and partial 5′ and 3′ non-coding region sequences. We uploaded the acquired sequence to GenBank (Accession Number, MK855081). The functional domain of HMGB1 was predicted by SMART software. Like HMGB1 in mammals, duck HMGB1 has two functional domains: BoxA and BoxB (Figure 1A). The phylogenetic tree was constructed with full-length HMGB1 protein and indicated three major branches: mammals, fish, and birds. DuHMGB1 was branched with birds and showed higher evolutionary relationship than with mammals and fish (Figure 1B). The alignment of multiple sequences generated by ClustalW2 showed that the duHMGB1 displayed high sequence identity with HMGB1 of chicken (99%), human (89%), or mouse (89%), suggesting that HMGB1 is highly conserved across species (Figure 1C).

### Tissue distribution of HMGB1 in healthy ducks

**Figure 2:**
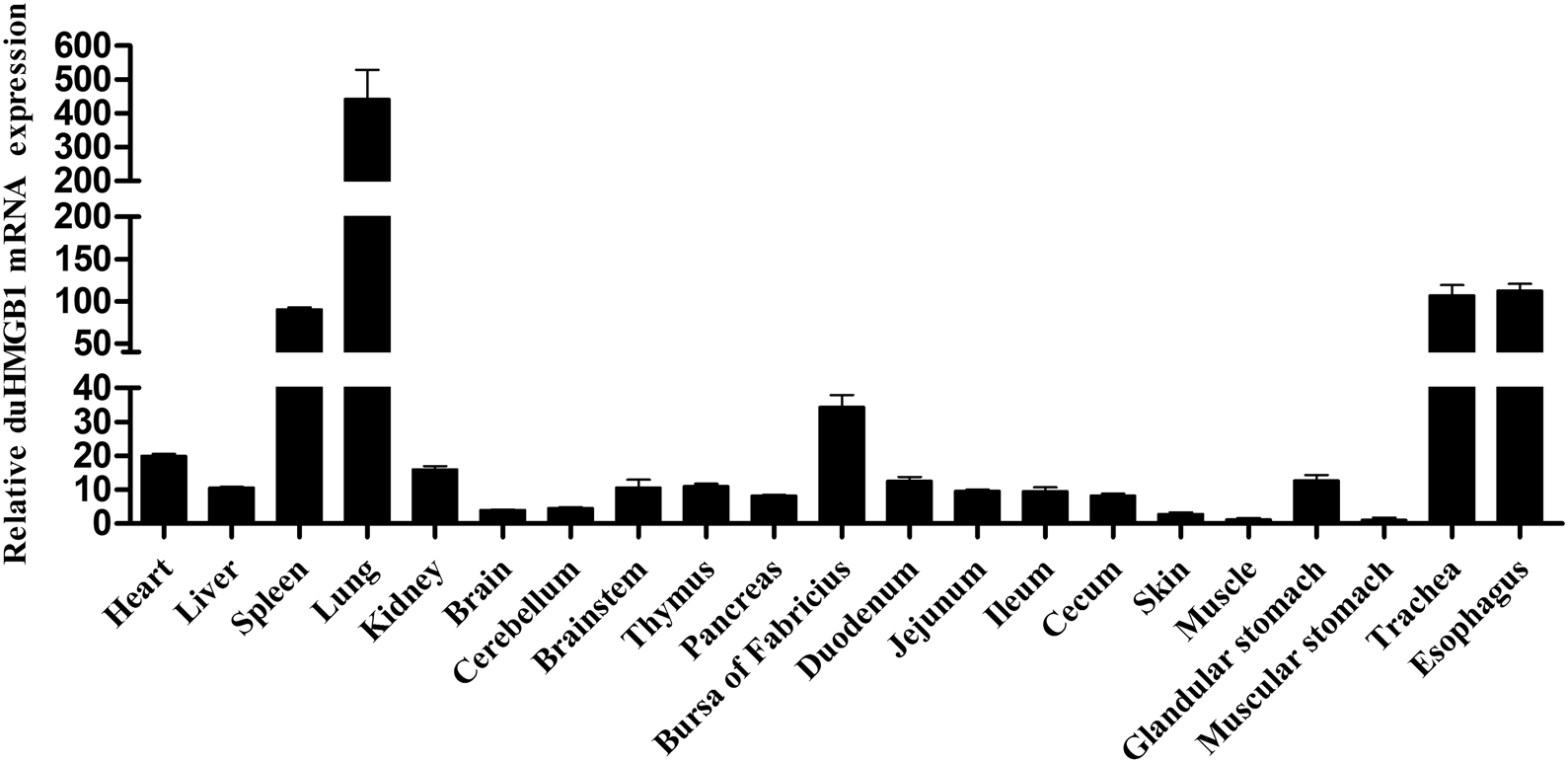
**Tissue distribution of duHMGB1 transcripts in healthy Cherry Valley ducks**. The relative mRNA levels were normalized to the expression of the GAPDH gene from various tissues. Each result represented the expression level of HMGB1 relative to the muscular stomach in the test tissue. Data are represented as the mean value ± SE of three experiments.

QRT-PCR detected the expression level of duHMGB1 in 21 tissues. Figure 2 shows that duHMGB1 was expressed in all tested tissues especially in the spleen, trachea, and esophagus; the highest expression was found in the lung. However, expression was weak in the brain, cerebellum, skin, muscle, and muscle stomach. The results showed that HMGB1 was expressed in more than 20 tissues, indicating that this factor can play a role in multiple tissues.

### duHMGB1 is expressed in the nucleus, but transfers to the cytoplasm upon LPS stimulation

**Figure 3:**
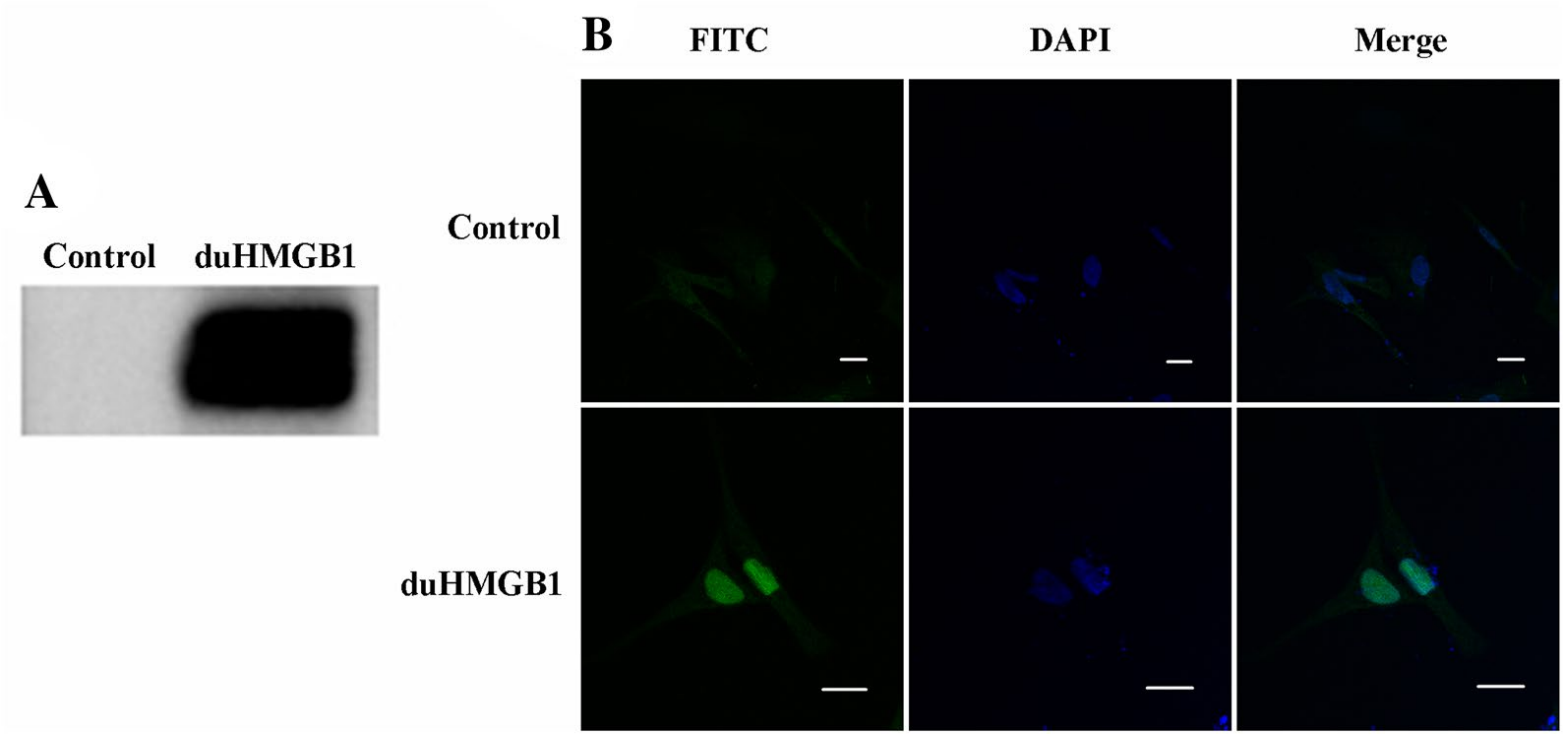
**duHMGB1 is localized in the nucleus under normal condition**. **A** Expression of recombinant pcDNA3.0(+)-duHMGB1 in DEF cells after transfection as shown by western blotting using anti-Flag antibody; **B** Sub-cellular localization of over-expressed duHMGB1 as shown by immunofluorescence. duHMGB1 plasmid was transfected in DEF cells for 24 h. Indirect immunofluorescence was performed using mouse anti-Flag antibody and FITC-labelled goat anti-mouse IgG (green). Nuclei were counterstained in blue (DAPI). All lines represent 25 μm length.

**Figure 4:**
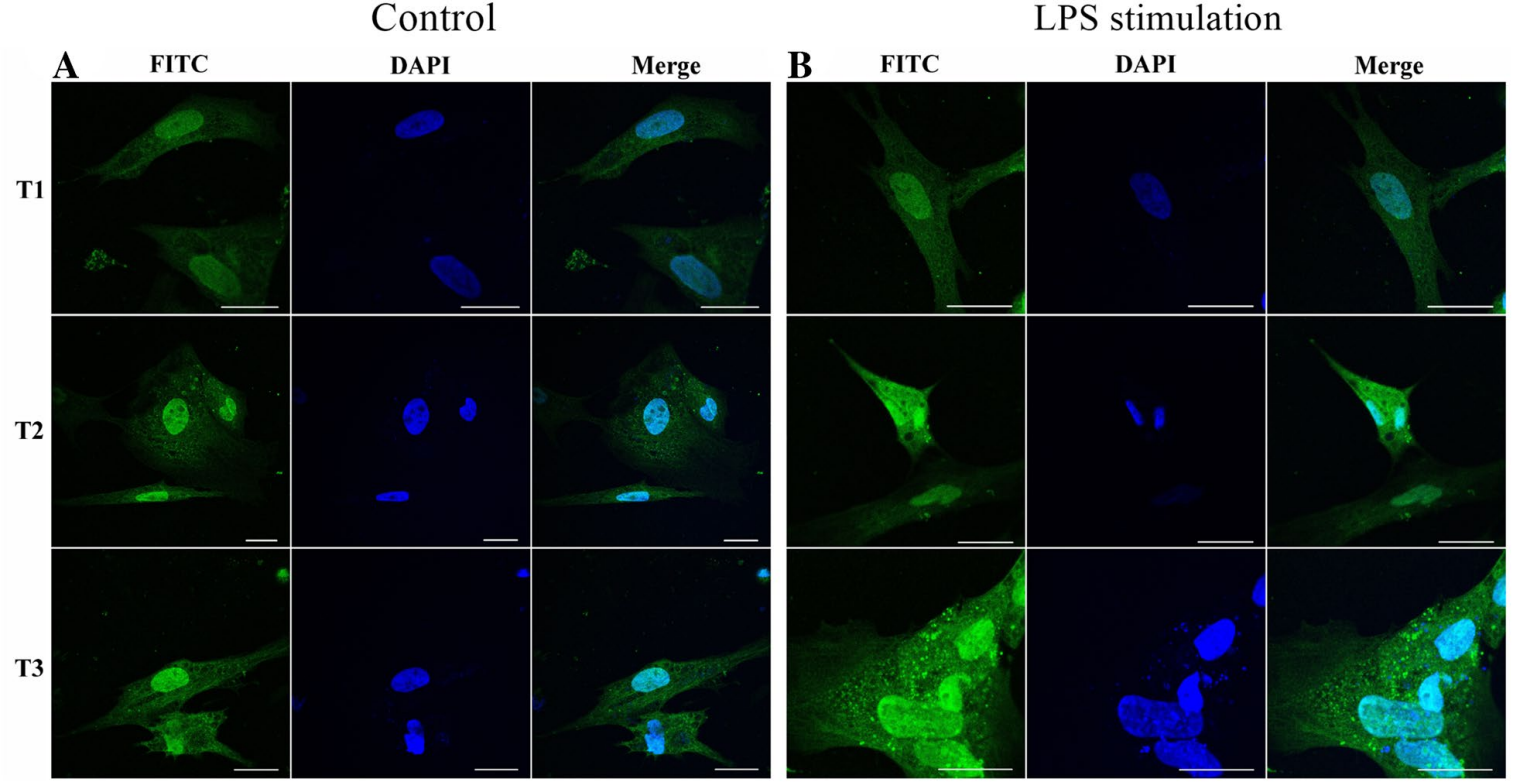
**LPS stimulation induces transfer of over-expressed recombinant duHMGB1 from nucleus to cytoplasm**. Immunofluorescence imaging was performed without LPS stimulation (control group) (A) and after LPS stimulation (**B**). DEF cells were transfected with pcDNA3.0(+)-duHMGB1-Flag plasmid for 24 h. Indirect immunofluorescence was performed after staining with mouse anti-Flag antibody and FITC-labelled goat anti-mouse IgG (green) 12 h (T1), 24 h (T2) and 36 h (T3) after LPS stimulation. Nuclei were counterstained in blue (DAPI). All lines represent 25 μm length.

The results of indirect immunofluorescence and western blot analyses demonstrated that recombinant duHMGB1 plasmid was expressed in DEF cells (Figure 3). Indirect immunofluorescence showed that duHMGB1 was mostly expressed in the nucleus after the plasmid was transfected into cells for 24 h (Figure 3B). The expression of duHMGB1 obviously increased in the cytoplasm after LPS stimulation of DEF cells at 24 h versus the control group (Figure 4).

### duHMGB1 is modestly involved in the process of apoptosis

**Figure 5:**
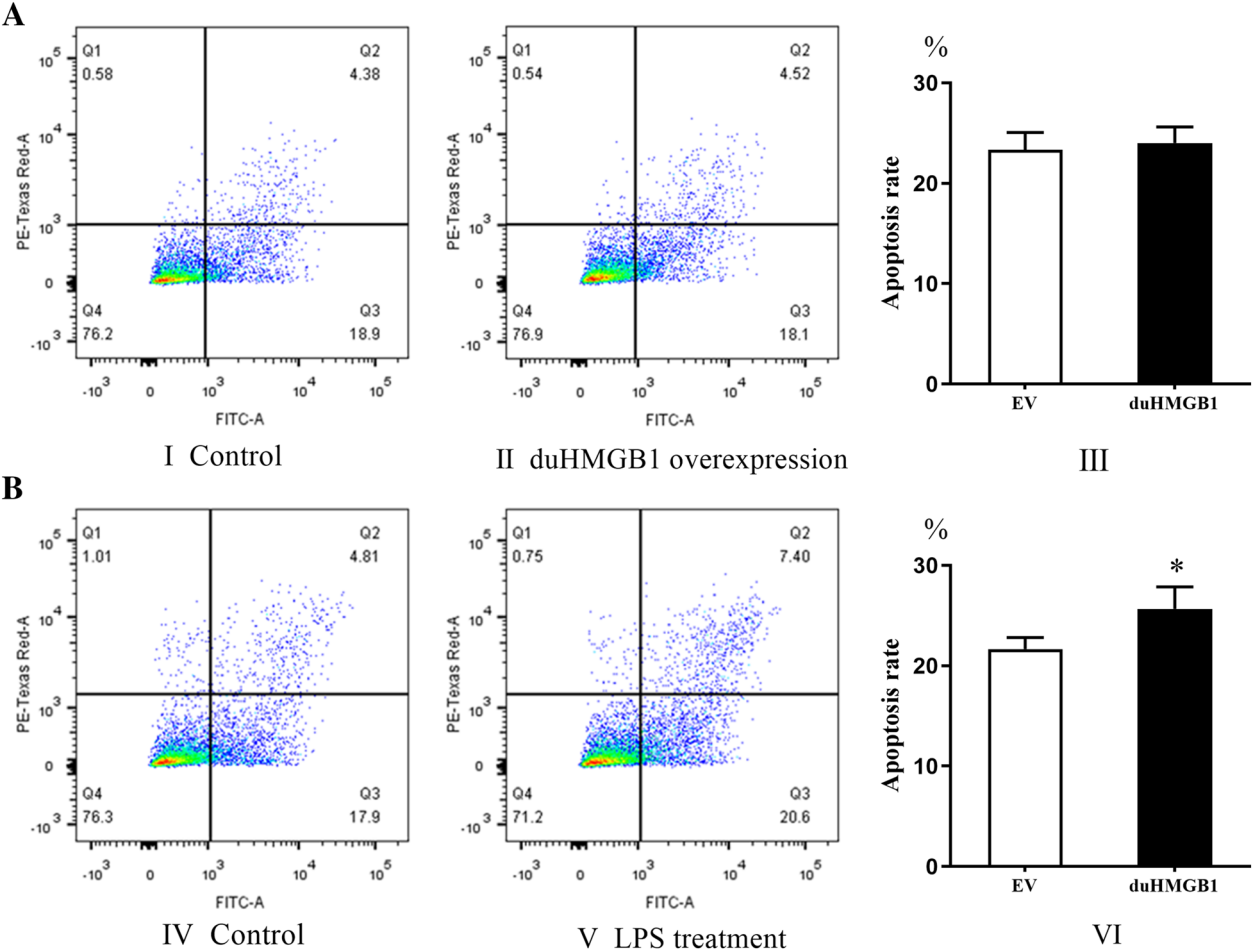
**duHMGB1 overexpression in DEF cells favours apoptosis only after LPS activation**. **A** No effect of duHMGB1 overexpression on spontaneous apoptosis. DEF cells were transfected with duHMGB1 plasmid or empty plasmid (control group). Apoptosis was analyzed by flow cytometry using PI (y axis) and FITC-conjugated annexin V (x axis) after additional 48 h of culture. **B** Effect of duHGMB1 overexpression on apoptosis after LPS stimulation. DEF cells were transfected with duHMGB1 plasmid or empty plasmid (control group), After 24 h, 500 ng/mL LPS was added and the culture was continued for 24 h. The cells were analyzed by flow cytometry for PI (y axis) and FITC-conjugated annexin V (x axis). The total percentages of PI^−^ annexin V^+^ cells (Q3) and PI^+^annexin V^+^ cells (Q2) indicate the apoptosis rate. I, II, IV and V are from a single experiment, which was representative of three separately performed experiments. The bar graphs (III and VI) mean value ± SE of three experiments. Mann–Whitney U test was performed to evaluate the differences. *P < 0.05.

Our results showed that duHMGB1 overexpression had no effect on apoptosis in DEF cells (Figure 5A, B) shows that overexpression of duHMGB1 promoted apoptosis of DEF cells when induced with LPS versus the control group; this pro-apoptotic effect is modest. The apoptosis rate induced by LPS in the experimental group was 1.2-fold higher than in the control group (*P *< 0.05). We also found that knocking-down of duHMGB1 gene expression in DEF cells was achieved using siRNA interference (Additional file 4A) without altering the apoptosis rate after 24 h of culture (Additional file 4B). These results indicate that duHMGB1 is modestly involved in apoptosis.

### duHMGB1 is involved in innate immunity

**Figure 6:**
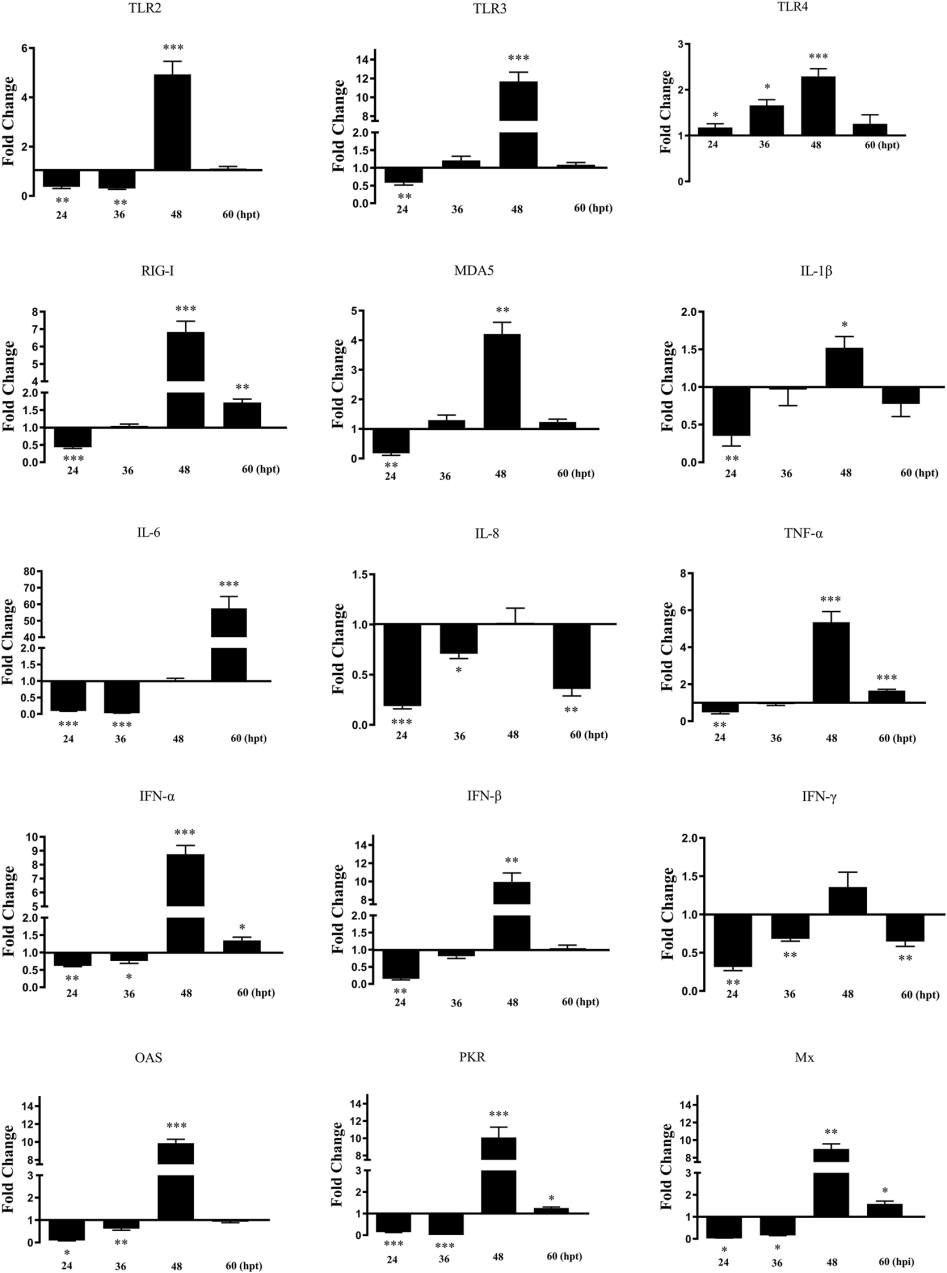
**Overexpression of duHMGB1 induces gene expression of pattern recognition receptors, pro-inflammatory cytokines and anti-viral molecules in DEF cells**. The experimental group was DEF cells transfected with duHMGB1, and the control group was DEF cells transfected with empty vector. Cells were collected at 24, 36, 48 and 60 hpt analyzing inducible gene expressions using qRT-PCR. Fold-changes in gene expression were calculated using the 2^−ΔΔCT^ method with GAPDH serving as a normalization gene and mean control values as baseline reference. Data are represented as the mean value ± SE of three experiments. Mann–Whitney U test was performed to evaluate the differences. *P < 0.05; **P < 0.01; ***P < 0.001.

To investigate the role of duHMGB1 in duck innate immunity, the pcDNA3.0(+)-duHMGB1-Flag or empty vector were transfected into DEF cells. The changes in mRNA expression of five pattern recognition receptors (TLR2, TLR3, TLR4, RIG-I, and MDA5), four proinflammatory cytokines (IL-1β, IL-6, IL-8, and TNF-α), three interferons (IFN-α, IFN-β, and IFN-γ), and ISGs (PKR, OAS, and Mx) were detected by qRT-PCR. Figure 6 shows that expression of all genes was mostly downregulated until up to 36 hpt and then upregulated at 48 hpt for PRRs such as TLR2, TLR4, TLR3, RIG-I and MDA5, for pro-inflammatory cytokines such as IL-1β and TNF-α, interferons such as IFN-α and IFN-β, and anti-viral molecules (OAS, PKR and Mx). The IL-6 response was significantly induced after 60 hpt.

**Figure 7:**
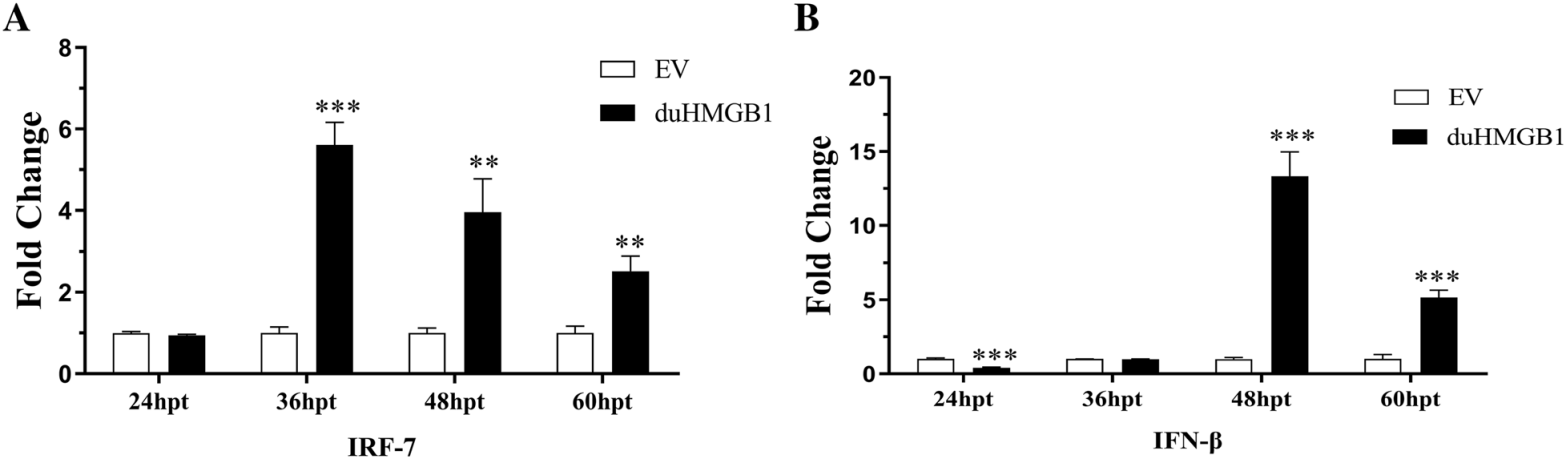
**Overexpression of duHMGB1 activates the IFN-I signaling pathway**. A dual luciferase reporter gene assay was used to study the IFN-I signaling pathway. pcDNA3.0(+)-duHMGB1-Flag and empty vector (control group) plasmids (500 ng/well) were co-transfected with reporter plasmids (100 ng/well) (**A**) pGL3-IRF7; (**B**) PGL3-IFN-β with pRL-TK (normalization) (50 ng/well). After 36 hpt, cells were harvested, and luciferase activity was measured. Relative IRF-7-, or IFN-β-reporter activation was calculated as fold-change in normalized Firefly luciferase activity with reference to mean control values set to 1. Data were means from three independent experiments and each experiment was analyzed in triplicate. Mann–Whitney U test was performed to evaluate the differences. *P < 0.05; **P < 0.01; ***P < 0.001.

The pcDNA3.0(+)-duHMGB1-Flag plasmid and reporter plasmids were co-transfected into DEF cells for a luciferase reporter assay to further demonstrate that duHMGB1 is involved in the signaling pathway of IFN-β in DEF cells. Figure 7 shows that duHMGB1 significantly activated IFN-β and IRF-7 luciferase activities versus empty vectors (13.3-fold at 48 hpt, *P* < 0.001; 5.6-fold at 36 hpt, *P* < 0.001). Overexpression of duHMGB1 in DEF cells had no significant effect on the NF-κB promoter activity (data not shown).

### duHMGB1 has broad-spectrum anti-viral activity

**Figure 8:**
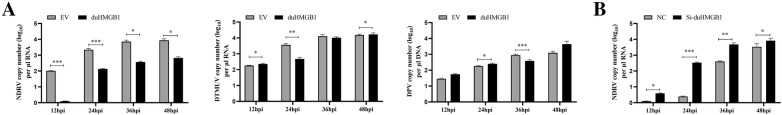
**duHMGB1 mediates anti-viral activity**. **A** duHMB1 overexpression induced inhibition of viral replication for NDRV, DTMUV and DPV. The duHMGB1 vector (pcDNA3.0-duHMGB1-Flag) and the empty vector (EV, control group) were transfected for 24 h. Then cells were infected with viruses at a dose of 10 TCID_50_/mL; **B** duHMGB1 knocking-down induced enhanced replication of NDRV. Si-duHMGB1-2 and Si-NC (control group) were transfected in DEF cells for 36 h. Then cells were infected with NDRV at 1 TCID_50_/mL. The culture supernatants (same sentence for A and B) were collected for detecting the viral titers at 12, 24, 36 and 48 hpi by RT-qPCR. Viral copy number was expressed as copy number (log_10_) per µL RNA or DNA related to the virus. Mann–Whitney U test was performed to evaluate the differences. *P < 0.05; **P < 0.01; ***P < 0.001.

The significant changes in the mRNA expression levels of IFN-α, β, γ, and ISGs after overexpression of duHMGB1 suggest that duHMGB1 has good antiviral effects at later stages. Cells transfected with pcDNA3.0(+)-duHMGB1-Flag or empty vector were infected with NDRV, DPV, or DTMUV. The changes in RNA or DNA expression of the three viruses were measured by qRT-PCR to confirm the antiviral function of duHMGB1. Figure 8 shows that the RNA expression of NDRV was decreased by 16.4-fold (*P* < 0.001) at 24 hpi versus the control group. By contrast, knocking-down duHMGB1 using siRNA interference showed increase of NDRV replication. In addition, duHMGB1 displayed the strongest anti-virus infection ability against DTMUV at 24 hpi among the four scheduled time points. Versus the control group, the RNA expression of virus decreased 8.2-fold (*P* < 0.01). Figure 8 shows that the DNA expression of DPV was down-regulated by 2.3-fold (*P* < 0.001) versus the control group at 36 hpi. In summary, HMGB1 displayed antiviral effects on a single-stranded RNA virus (DTMUV), double-stranded segmental RNA virus (NDRV), and DNA virus (DPV); thus, HMGB1 possesses broad-spectrum antiviral function.

### duHMGB1 impacts antiviral and innate immune responses after NDRV infection

**Figure 9:**
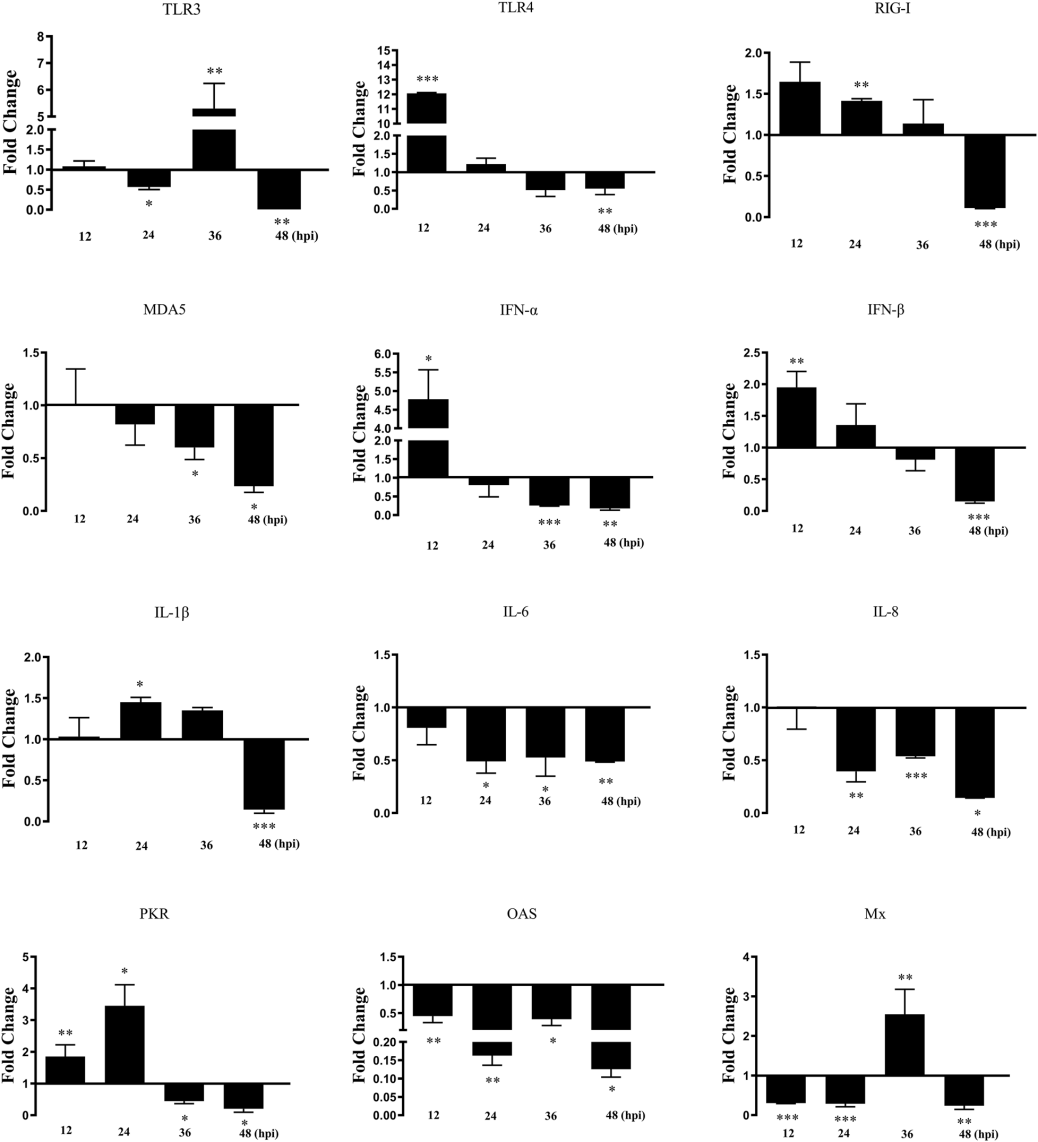
**duHMGB1 over-expression in DEF cells modulates gene expression pattern of pattern recognition receptors, cytokines and anti-viral molecules after NDRV infection**. The experimental group was DEF cells transfected with duHMGB1, and the control group was DEF cells transfected with empty vector. After 24 h transfection, the cells were infected with NDRV at 10 TCID_50_/mL. Cells were collected at 24, 36, 48 and 60 hpi analyzing inducible gene expressions using qRT-PCR. Fold-changes in gene expression were calculated using the 2^−ΔΔCT^ method with GAPDH serving as a normalization gene and mean control values as baseline reference. Data are represented as the mean value ± SE of three experiments. The differences among the groups were evaluated by Nonparametric tests (Mann–Whitney U tests) using SPSS software version 17.0 (SPSS Inc., Chicago, IL, USA), *P < 0.05; **P < 0.01; ***P < 0.001.

DEF cells were stimulated by NDRV at 24 h after overexpression of duHMGB1 to explore the change of antiviral and innate immune responses after NDRV infection. Figure 9 shows that the mRNA expression levels of RIG-I receptors, IFN-β, IFN-γ, and PKR associated with antiviral response were up-regulated both at 12 and 24 hpi versus the control group. The mRNA expression level of IFN-α was up-regulated by 4.8 times (*P* < 0.05) at 12 hpi versus the control group. These results suggest that duHMGB1 cooperates with RIG-I receptor to recognize NDRV and thus promote the expression of interferon and PKR. It has obvious antiviral effects at 12 and 24 hpi.

**Figure 10:**
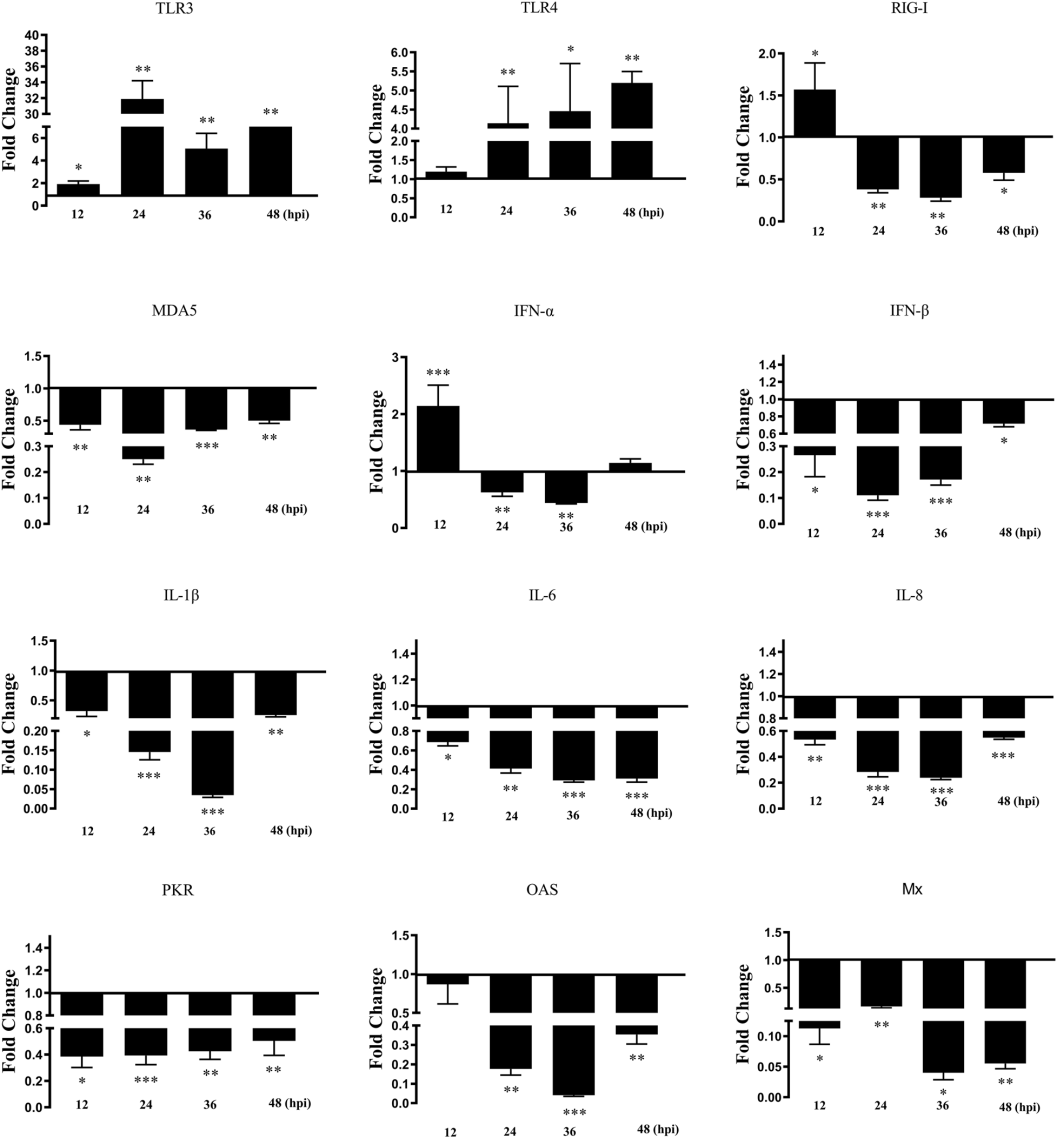
**Knocking-down duHMGB1 expression reduces or suppresses induction of some major innate immune and anti-viral gene expression after NDRV infection**. The experimental group was DEF cells transfected with Si-duHMGB1, and the control group was DEF cells transfected with Si-NC. After 36 h transfection, the cells were infected with NDRV at 1 TCID_50_/mL. Cells were collected at 24, 36, 48 and 60 hpi for analyzing inducible gene expressions using qRT-PCR. Fold-changes in gene expression were calculated using the 2^−ΔΔCT^ method with GAPDH serving as a normalization gene and mean control values as baseline reference. Data are represented as the mean value ± SE of three experiments. The differences among the groups were evaluated by Nonparametric tests (Mann–Whitney U tests) using SPSS software version 17.0 (SPSS Inc., Chicago, IL, USA), *P < 0.05; **P < 0.01; ***P < 0.001.

To further verify this hypothesis, three interfering RNAs of duHMGB1 were designed: Figure 5A shows that Si-HMGB1-2 displayed the highest interference efficiency. Therefore, Si-HMGB1-2 was selected as the interfering RNA for subsequent experiments. Figure 10 shows that the mRNA expression levels of IFN-β and PKR were down-regulated at 12 and 24 hpi versus the control group. The expression levels of RIG-I and IFN-α were down-regulated at 24 hpi versus the control group—RIG-I was down-regulated 2.6-fold (*P* < 0.01). These results indicated that the expression pattern of the genes above in HMGB1-knockdown cells was roughly opposite of that in HMGB1-overexpressing cells during NDRV infection.

## Discussion

HGMB1 is a highly conserved protein present everywhere from yeasts, bacteria, plants, invertebrates to mammals [25–29]. More specifically, HMGB1 has been demonstrated to be involved in immune responses to infection, injury, and inflammation in mammals [1]. We have cloned and sequenced duHMGB1 from the cherry valley duck. We found that duHMGB1 has the highest sequence identity with chicken HMGB1 (99%). However, the identity was also very high between duck and human. Moreover, duHMGB1 gene expression was found to be widely distributed in duck tissues. This is consistent with the widespread distribution of HMGB1 in different mammalian and chicken tissues. However, the content of HMGB1 in lymphoid tissues and testis of mammals is higher [30], the content of HMGB1 in ileum and bursa of fabricius is higher in chickens [31], and the content of HMGB1 is highest in lung tissues of ducks.

Analysis of the sequence showed that duHMGB1 has two nuclear localization sequences like mammalian HMGB1 [32]. We observed that overexpressed recombinant flagged duHMGB1 after transfection of DEF cells localized mostly to the nucleus (as in mammals, [3]). However, duHMGB1 was released from the nucleus to cytoplasm as soon as 24 h post-stimulation with LPS. Extensive acetylation of HMGB1 upon activation by LPS may be a hypothetic mechanism since HMGB1 acetylation is induced by LPS in mammalian cells and since this acetylation is the signal to induce relocation of nuclear HMGB1 to cytoplasm [3].

The role of duHMGB1 in apoptosis was not clearly observed in DEF cells after overexpression or gene knocking-down using RNA interference, at variance with the situation observed in mammals [33]. However, after LPS stimulation, duHMGB1 overexpressing DEF cells had an increased apoptotic rate compared to empty vector transfected control cells. In mammals, HMGB1 undergoes a redox reaction in the extracellular environment to induce apoptosis through the mitochondrial pathway [34, 35]. The reason, according to the literature, may be that LPS induces the transfer of HMGB1 from the nucleus, but also the release of the protein in the extracellular environment. The biological functions of HMGB1 are determined by the post-translational modifications of the protein (acetylation, etc.) in addition to its subcellular localization [36]. We may thus suspect that LPS is able to induce HMGB1 release from DEF cells in supernatant, as in mammals. Nevertheless, for DEF cells, it is a hypothesis that would need to be confirmed using Western blotting or ELISA.

Our results show that overexpression of duHMGB1 in DEF cells induced a strong timely expression of TLR (TLR2, TLR4, TLR3) and PRRs (MDA5 and RIG-I) as well as interferons type I, anti-viral molecules (PKR, OAS, and Mx) and pro-inflammatory cytokines (IL-1β, IL-6, IL-8, and TNF-α). This indicates that HMGB1 can induce a clear pattern of gene expression linked to inflammatory and anti-viral innate immune responses in DEF cells. In addition, we demonstrated that duHMGB1 overexpression in DEF cells can activate the IFN-I signaling pathway, which is similar but not identical to mammals and chickens. HMGB1 in mammals can interact with TLRs and activate related signal transduction pathways to produce a range of cytokines [36]. Qu et al. reported that chicken HMGB1 is a significant inflammation factor in NDV infection. Chicken HMGB1 is involved in NDV-induced NF-κB activation and the inflammatory response, and promotes inflammatory cytokine production through the RAGR, TLR2, and TLR4 receptors [31]. Our results indicate that the expression of TLR4 and RIG-I were up-regulated after duHMGB1 overexpression. There may be molecular cooperative relationships between duHMGB1 and TLR4, duHMGB1 and RIG-I. However, the functional cooperation between them requires further research before firm conclusions are reached about an antiviral mechanism.

Although the previous results suggest that HMGB1 has no effect on PRRSV and NDV replication, HMGB1 promotes virus-induced NF-κB activation and subsequent expression of inflammatory cytokines, enhances the efficiency of virus-induced inflammatory responses [16, 31]. Thus, we tried to test the potential antiviral effect of duHMGB1 by comparing the viral load between duHMGB1 overexpression and knockdown in DEF cells, which showed an antiviral effect of duHMGB1 on DTMUV, NDRV and DPV (the infectious diseases caused by these three viruses has resulted in massive economic loss to the duck industry) [37–39]. Since the effect of duHMGB1 overexpression has been the most effective to inhibit viral replication of NDRV at all time-points (from 12 to 48 hpi), the immune response in DEF cells was subsequently studied after NDRV infection as an example.

By comparing the effects of duHMGB1 overexpression and knockdown in DEF cells with NDRV infection on immune response, the molecules that may be related to the antiviral and inflammatory responses were RIG-I, IFN-β, PKR and IL-1β. It was reported that NDRV could be cooperatively recognized by several pathogen recognition receptors that initiate innate immunity [19], which was similar to our results. In mammals, viral replication is sensed by RIG-I to initiate the cascade of events leading to the activation of transcription factors, IRF-3/-7 and NF-κB, and the activation of IFN genes. The primary function of the IFN system is to sense non-self RNA and to eradicate the invading RNA, which includes RNA derived from the replication of DNA viruses. IFNs induce the transcriptional activation of PKR, activated by double-stranded RNA, to restrict viral replication by phosphorylating the protein synthesis initiation factor eIF-2α and reduce levels of viral protein synthesis [40]. Besides, Chen et al. reported that duck IRF-7 can activate the IFN-β promoter to induce type I interferon (IFN-α and IFN-β) transcription, inhibiting DTMUV replication in vitro [41]. Tang et al. found that HMGB1 and IL-6 are involved in inflammation caused by *Pasteurella multocida* infection in chickens [42]. The physiological effects of these molecules in birds are similar to those in mammals.

In summary, we cloned duHMGB1 for the first time and found that duHMGB1 is widely distributed in the tissues of the Cherry Valley duck and can be released from the nucleus into the cytoplasm during LPS stimulation. Our results also demonstrate that duHMGB1 has broad-spectrum antiviral activity and induces the expression of antiviral proteins in DEF cells, which provide a new insight into understanding how HMGB1 mediates innate antiviral immunity responses in ducks. These discoveries will hopefully provide a potential therapeutic target against NDRV.

## Supplementary information

**Additional file 1: Primer sequences used in this study for gene cloning.**

**Additional file 2: Reference sequences information.**

**Additional file 3: Primer sequences used in this study for qRT-PCR.**

**Additional file 4: Knocking-down of duHMGB1 gene expression in DEF has no effect on apoptosis**. **A** RNA interference efficiency: the interference efficiency of duHMGB1-2 is 82.2%. siRNA for duHMGB1 and siRNA control were transfected, and DEF cells were cultured for 36 h. duHMGB1 expression levels were normalized to the GAPDH gene and calculated using the 2^−ΔΔCt^ method. Data are represented as the mean value ± SE of three experiments; **B** Effect of duHMGB1 interference in DEF cells on apoptosis. Apoptosis was analyzed, 48 h after siRNA transfection, by flow cytometry using PI (y axis) and FITC-conjugated annexin V (x axis). The total percentages of PI^−^ annexin V^+^ cells (Q3) and PI^+^ annexin V^+^ cells (Q2) indicate the apoptosis rate. I and II are from a single experiment, which was representative of three separately performed experiments. The bar graphs (III) mean value ± SE of three experiments. Mann–Whitney U test was performed to evaluate the differences. *P < 0.05; **P < 0.01; ***P < 0.001.

## Data Availability

We have uploaded the acquired sequence of duHMGB1 to GenBank (accession number, MK855081), and other datasets analyzed during the current study are available from the corresponding author on reasonable request.

## Abbreviations

HMGB1: high-mobility group box 1 protein
DAMPs: damage-associated molecular patterns
duHMGB1: duck HMGB1
DEF: duck embryo fibroblast
NDRV: novel duck reovirus
LPS: lipopolysaccharide
DTMUV: duck Tembusu virus
DPV: duck plague virus
TCID_50_: 50% tissue culture infective dose
PVDF: polyvinylidene fluoride
FITC: fluorescein isothiocyanate
qRT-PCR: quantitative real-time PCR
hpt: hours post-transfection
hpi: hours post-infection
SE: standard error
EV: empty vector
