# Supplementary material for: High-mobility group box 1 protein (HMGB1) from Cherry Valley duck mediates signaling pathways and antiviral activity
Source: Vet Res. 2020 Feb 18;51:12. doi: 10.1186/s13567-020-00742-8 (PMC7027276; doi:10.1186/s13567-020-00742-8)
Supplement: Supplementary file 1 — Additional file 1: Primer sequences used in this study for gene cloning. [file 13567_2020_742_MOESM1_ESM.docx]

**Additional file 2 Primer sequences used in this study for gene cloning.**

| **Primer name** | **Primer sequence (5′-3′)** | **Purpose** |
| --- | --- | --- |
| gHMGB1-F1 | CCACACGGAAAATCATCAAA | Gene cloning |
| gHMGB1-R1 | TGTACCAGGCAAGGTTAGTGG |  |
| gHMGB1-F2 | AGTGTGAGGAGGCTGCGTAT | Gene cloning |
| gHMGB1-R2 | CGGCCTTGGCAACTACTTTT |  |
| rHMGB1-F | CTTGGTACCGAGCTCGGATCCGCCACCATGGGCAAAGGCGATCCTAAG | Gene cloning |
| rHMGB1-R | CTCTAGACTCGAGCGGCCGCTTATTCATCATCATCATCATC |  |
