# Supplementary material for: High-mobility group box 1 protein (HMGB1) from Cherry Valley duck mediates signaling pathways and antiviral activity
Source: Vet Res. 2020 Feb 18;51:12. doi: 10.1186/s13567-020-00742-8 (PMC7027276; doi:10.1186/s13567-020-00742-8)
Supplement: Supplementary file 2 — Additional file 2: Reference sequences information. [file 13567_2020_742_MOESM2_ESM.docx]

**Additional file 3 Reference sequences information.**

| **Species** | **GenBank accession number** |
| --- | --- |
| *Ailuropoda melanoleuca* | XM_011219124.2 |
| *Alligator sinensis* | XM_014527067.2 |
| *Anas platyrhynchos* | XM_027469875.1 |
| *Anser cygnoides* | XM_013189397.1 |
| *Callorhinchus milii* | NM_001292579.1 |
| *Chelonia mydas* | XM_007064778.2 |
| *Columba livia* | XM_013366679.2 |
| *Ctenopharyngodon idella* | JN866602.1 |
| *Danio rerio* | AY398358.1 |
| *Equus caballus* | NM_001081835.2 |
| *Gallus* | NM_204902.2 |
| *Geospiza fortis* | XM_005426371.1 |
| *Gorilla* | XM_019039569.1 |
| *Homo* | CR456863.1 |
| *Lctalurus punctatus* | XM_017491680.1 |
| *Oncorhynchus mykiss* | DQ403261.1 |
| *Oryctolagus cuniculus* | XM_008273516.2 |
| *Papio Anubis* | XM_009191721.3 |
| *Pelodiscus sinensis* | XM_006125307.2 |
| *Python bivittatus* | XM_007424365.2 |
