# Supplementary material for: High-mobility group box 1 protein (HMGB1) from Cherry Valley duck mediates signaling pathways and antiviral activity
Source: Vet Res. 2020 Feb 18;51:12. doi: 10.1186/s13567-020-00742-8 (PMC7027276; doi:10.1186/s13567-020-00742-8)
Supplement: Supplementary file 3 — Additional file 3: Primer sequences used in this study for qRT-PCR. [file 13567_2020_742_MOESM3_ESM.docx]

**Additional file 4 Primer sequences used in this study** **for** **qRT-PCR.**

| **Primer name** | **Primer sequence (5′-3′)** | **Product size (bp)** | **GenBank no.** |
| --- | --- | --- | --- |
| qHMGB1-F | TTTGCTCTGAGTTTCGTCCA | 142 | MK855081 |
| qHMGB1-R | TTCAGTTTGGCAGCCTTTTT |  |  |
| qTLR2- F | AAGAAAATGGAGCTGCTGGA | 231 | KX687002.1 |
| qTLR2-R | GAAAAACACAGCGCAGATCA |  |  |
| qTLR3-F | GAGTTTCACACAGGATGTTTAC | 201 | KU949327.1 |
| qTLR3-R | GTGAGATTTGTTCCTTGCAG |  |  |
| qTLR4-F | ACCCATTGTCACCAACATCATC | 195 | NM_001310413.1 |
| qTLR4-R | TGCCTCAGCAAGGTCTTATTCA |  |  |
| qRIG-I-F | GCTACCGCCGCTACATCGAG | 224 | KP981415.1 |
| qRIG-I-R | TGCCAGTCCTGTGTAACCTG |  |  |
| qMDA5-F | GCTACAGAAGATAGAAGTGTCA | 120 | KF709945.1 |
| qMDA5-R | CAGGATCAGATCTGGTTCAG |  |  |
| qIL-1β-F | TCATCTTCTACCGCCTGGAC | 149 | DQ393268.1 |
| qIL-1β-R | GTAGGTGGCGATGTTGACCT |  |  |
| qdIL-6-F | TTCGACGAGGAGAAATGCTT | 150 | JQ728554.1 |
| qdIL-6-R | CCTTATCGTCGTTGCCAGAT |  |  |
| qdIL-8-F | AAGTTCATCCACCCTAAATC | 182 | NM_001310420.1 |
| qdIL-8-R | GCATCAGAATTGAGCTGAGC |  |  |
| qTNF-α-F | GAAGGGAATGAACCCTCCTC | 89 | EU375296.1 |
| qTNF-α-R | CAGGTTGCTGCACATACACC |  |  |
| qIFN-α-F | TCCTCCAACACCTCTTCGAC | 232 | KF731866.1 |
| qIFN-α-R | GGGCTGTAGGTGTGGTTCTG |  |  |
| qIFN-β-F | AGATGGCTCCCAGCTCTACA | 210 | KM035791.2 |
| qIFN-β-R | AGTGGTTGAGCTGGTTGAGG |  |  |
| qIFN-γ-F | GCTGATGGCAATCCTGTTTT | 247 | KF746067.1 |
| qIFN-γ-R | GGATTTTCAAGCCAGTCAGC |  |  |
| qOAS-F | TCTTCCTCAGCTGCTTCTCC | 187 | KY775584.1 |
| qOAS-R | ACTTCGATGGACTCGCTGTT |  |  |
| qPKR-F | AATTCCTTGCCTTTTCATTCAA | 118 | KR025553.1 |
| qPKR-R | TTTGTTTTGTGCCATATCTTGG |  |  |
| qMx-F | TGCTGTCCTTCATGACTTCG | 153 | KR025554.1 |
| qMx-R | GCTTTGCTGAGCCGATTAAC |  |  |
| qGAPDH-F | ATGTTCGTGATGGGTGTGAA | 176 | GU564233.1 |
| qGAPDH-R | CTGTCTTCGTGTGTGGCTGT |  |  |
| qDTMUV-F | CGCTGAGATGGAGGATTATGG | 225 | KP096415.1 |
| qDTMUV-R | ACTGATTGTTTGGTGGCGTG |  |  |
| qNDRV-F | TGAGTGGCTGGGAACTGT | 233 | JX826587.1 |
| qNDRV-R | CCATAAAGGAAGCAGAAG |  |  |
| qDPV-F | GCTTCACCTGCCCGGTCAC | 113 | JQ647509.1 |
| qDPV-R | CCACTGTCGGCACATCTAGCA |  |  |
