# Supplementary figures and images for: High-mobility group box 1 protein (HMGB1) from Cherry Valley duck mediates signaling pathways and antiviral activity
Source: Vet Res. 2020 Feb 18;51:12. doi: 10.1186/s13567-020-00742-8 (PMC7027276; doi:10.1186/s13567-020-00742-8)

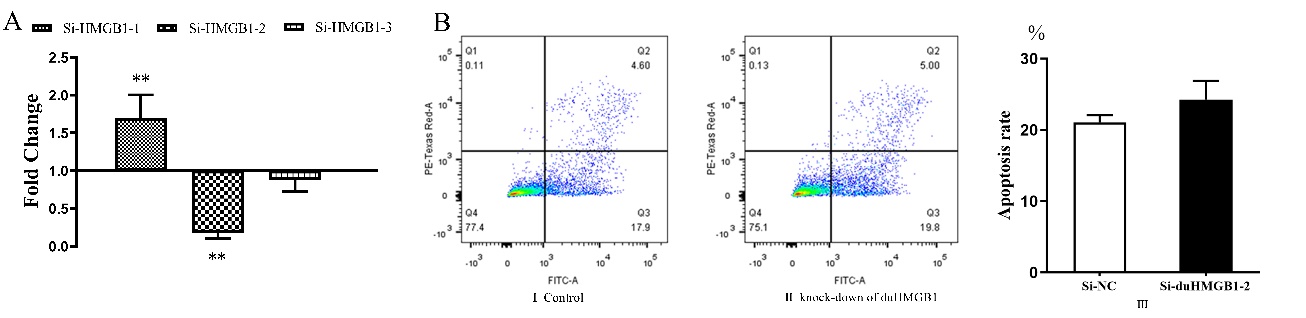

Supplement: Supplementary file 4 — Additional file 4: Knocking-down of duHMGB1 gene expression in DEF has no effect on apoptosis. A RNA interference efficiency: the interference efficiency of duHMGB1-2 is 82.2%. siRNA for duHMGB1 and siRNA control were transfected, and DEF cells were cultured for 36 h. duHMGB1 expression levels were normalized to the GAPDH gene and calculated using the 2−ΔΔCt method. Data are represented as the mean value ± SE of three experiments; B Effect of duHMGB1 interference in DEF cells on apoptosis. Apoptosis was analyzed, 48 h after siRNA transfection, by flow cytometry using PI (y axis) and FITC-conjugated annexin V (x axis). The total percentages of PI− annexin V+ cells (Q3) and PI+ annexin V+ cells (Q2) indicate the apoptosis rate. I and II are from a single experiment, which was representative of three separately performed experiments. The bar graphs (III) mean value ± SE of three experiments. Mann–Whitney U test was performed to evaluate the differences. *P < 0.05; **P < 0.01; ***P < 0.001. [file 13567_2020_742_MOESM4_ESM.docx]
